# Verbascoside targets endothelial HIF-1α/ Lysyl oxidase signaling to attenuate glomerular injury in diabetic nephropathy

**DOI:** 10.1080/13510002.2025.2598110

**Published:** 2025-12-08

**Authors:** Tianyu Kang, Bin Hou, Min Shi, Huan Liu, Yanan Li, Kaixin Li, Shuxin Li, Zetong Wu, Zhaopeng Xu, Mengnan Li

**Affiliations:** a Hebei Luoxue Innovation Medicine Research Institute, Shijiazhuang, People’s Republic of China; b College of Integrated Traditional Chinese and Western Medicine, Hebei Medical University, Shijiazhuang, People’s Republic of China; c State Key Laboratory for Innovation and Transformation of Luobing Theory, Shijiazhuang, People’s Republic of China; d Hebei Yiling Chinese Medicine Research Institute, Shijiazhuang, People’s Republic of China; e School of Pharmacy, Hebei University of Chinese Medicine, Shijiazhuang, People’s Republic of China

**Keywords:** Diabetic nephropathy, endothelial dysfunction, lysyl oxidase, HIF-1α signaling, mesangial proliferation, intercellular communication, natural medicines, oxidative stress

## Abstract

**Background:**

Diabetic nephropathy (DN) drives progressive renal fibrosis and functional decline, ultimately leading to end-stage renal disease. Pathological crosstalk between glomerular endothelial cells and mesangial cells is increasingly recognized as central to DN progression. However, whether endothelial-derived signaling specifically drives mesangial injury under diabetic conditions remains undefined.

**Methods:**

We applied multi-omics profiling to identify pathogenic drivers. Target validation included qPCR and immunofluorescence co-localization in renal tissues. *In vitro* endothelial-mesangial crosstalk was modeled using conditioned media (CM) from mouse GECs applied to mesangial cells. Verbascoside (VB) was screened via structure-based virtual docking against LOX/LOXL2 and binding affinity (KD) confirmed by biolayer interferometry (BLI). *In vivo* therapeutic efficacy of VB was assessed in db/db mice.

**Results:**

LOX/LOXL2 was robustly upregulated in diabetic endothelia. Inhibiting endothelial-derived LOX/LOXL2 or HIF-1α in GECs attenuated HG-induced mesangial dysfunction by reducing proliferation/viability, oxidative stress, and fibrosis. Mechanistically, HIF-1α drove LOX/LOXL2 expression. VB was identified as a novel dual LOX/LOXL2 inhibitor. VB-CM mitigated mesangial injury *in vitro*. VB treatment improved renal function, reduced oxidative damage, and ameliorated fibrosis.

**Conclusion:**

Endothelial HIF-1α/LOX signaling drives mesangial oxidative stress and fibrosis in DN. Verbascoside, a dual LOX/LOXL2 inhibitor, represents a promising therapeutic agent targeting this pathogenic axis.

## 1. Introduction

Diabetic kidney disease (DKD), a devastating microvascular complication afflicting up to 40% of diabetic patients, represents the primary pathway to end-stage renal failure [1]. Central to the glomerular injury process is endothelial cell (EC) dysfunction, manifesting not merely as passive structural damage but as pathological orchestration of intercellular crosstalk that drives disease progression [2–4]. Chronic hyperglycemia profoundly disrupts the complex signaling dialogue within the diabetic glomerular microenvironment, placing glomerular endothelial cells (GECs) at the center of injury amplification. Sustained high glucose subverts their essential paracrine functions, suppressing protective mediators vital for podocyte health while ramping up detrimental molecules that drive inflammation, fibrosis, and aberrant cell growth [5].

Pathogenic endothelial signaling dysfunction destabilizes the critical regulatory balance among glomerular endothelial cells (GEnCs), podocytes, and mesangial cells. Key mechanisms driving this imbalance include elevated secretion of latent TGF-β binding protein 1 (LTBP1) from diabetic ECs – which facilitates spatial targeting and activation of TGF-β complexes [6] – alongside disruption of NO-dependent paracrine crosstalk, where GEnC-derived nitric oxide normally induces cGMP formation in mesangial cells to modulate intraglomerular hemodynamics [7–9]. This breakdown in intercellular communication ultimately triggers a pathogenic cascade featuring uninhibited mesangial cell activation and proliferation, pathological extracellular matrix (ECM) deposition guided by aberrant GEnC signaling, structural damage and detachment of podocytes, and amplified inflammatory cell recruitment. Collectively, these events progress from dysfunctional endothelial signaling to overt glomerular deterioration, clinically manifesting as albuminuria, glomerulosclerosis, and irreversible loss of renal function.

Recent studies have also revealed that the dysfunction of glomerular endothelial cells (GEnCs) initiates profound alterations within the glomerular extracellular matrix (ECM), predominantly manifesting as progressive tissue stiffening [10,11]. Central to this pathological ECM remodeling is the aberrant activity of the lysyl oxidase (LOX) enzyme family, specifically Lysyl oxidase homolog 2 (LOXL2) [12,13]. These intracellular amine oxidases catalyze the formation of covalent cross-links within collagen and elastin fibers, thereby dramatically increasing the rigidity of the ECM infrastructure [14]. LOX enzyme family facilitates the advancement of both glomerulosclerosis and interstitial fibrosis [15–17]. This interaction underpins a potent LOX enzyme family-ROS amplification circuit, wherein ROS and associated inflammatory mediators induce the transcription factor HIF-1α [18–20], a key transcriptional activator that subsequently further upregulates gene expression of LOX family. While recognizing the central position of ECM stiffness in DKD progression, the specific contribution and potential as a pharmacologic target of LOX enzyme family originating from the endothelium itself in perturbing mesangial homeostasis remains inadequately defined.

Given the multi-factorial nature of DKD, natural compounds with polypharmacology and favorable safety profiles represent promising therapeutic avenues. Verbascoside, a principal phenylethanoid glycoside derived from Cistanche deserticola, demonstrates multimodal biological activities encompassing potent antioxidant, anti-inflammatory, and anti-fibrotic effects across organ systems [21–23]. Its renoprotective efficacy has been specifically validated in experimental models of glomerular injury and fibrosis, as established through well-documented studies [24,25]. Critically, while Verbascoside demonstrates established efficacy in kidney injury models, its specific impact on rescuing endothelial secretory networks and restoring healthy intercellular communication within the diabetic glomerulus remains largely uncharted territory.

This investigation employs an integrated experimental strategy to define the pathogenic contribution of endothelial LOX/LOXL2 in diabetic microvascular crosstalk and to substantiate Verbascoside as a mechanistically driven therapeutic candidate for DKD. Building on evidence of hyperglycemia-triggered endothelial LOX/LOXL2 overexpression, we provide causal evidence that GEnC-secreted LOX/LOXL2 enzymes directly induce mesangial cell dysregulation, driving oxidative stress, fibrosis, and collagen synthesis. This causal link was validated through targeted chemical inhibition of LOX/LOXL2 and suppression of the upstream regulator HIF-1α. Crucially, molecular modeling and Bio-Layer Interferometry (BLI) demonstrated that Verbascoside binds directly and with high affinity to both LOX and LOXL2 enzymes, confirming its mechanism as a dual natural inhibitor. Our study demonstrated that Verbascoside administration effectively alleviated key diabetic kidney disease manifestations in murine models, including reduced albuminuria, attenuated glomerulosclerosis, and diminished oxidative stress. These findings align with prior research showing Verbascoside's protective effects against kidney injury in various disease models [25–27]. Collectively, these findings establish endothelial LOX/LOXL2 as core regulators of pathogenic glomerular signaling in diabetes and position Verbascoside as an enzymatically targeted precision therapy for DKD acting through the disruption of this specific LOX/LOXL2-mediated axis.

## 2. Materials and methods

### 2.1. Chemicals and reagents

Cyasterone (Cat. No. AB1467), Diosmin (Cat. No. AB0713), Monnieriside G (Cat. No. AB1536), Astragalin (Cat. No. AB1957), Hyperin (Cat. No. AB0834), Tiliroside (Cat. No. AB1340), and Verbascoside (Cat. No. AB0497, 100–200 μM for *in vitro* studies) were obtained from Chengdu Alfa Biotechnology Co., Ltd. Rutin (Cat. No. ST03950120) and Ellagic acid (Cat. No. ST04700120) were obtained from Shanghai Standard Technology Co., Ltd. Isomartynoside (Cat. No. CFN97525) was obtained from ChemFaces Co., Ltd. Echinacoside (Cat. No. A0282) was obtained from MUST (Cheng Du) Biotechnology Co., Ltd. The pan lysyl oxidase inhibitor β-aminoproprionitrile (BAPN) (Cat. No. HY-Y1750), LOXL2 inhibitor PAT-1251 (Cat. No. HY-107422), and HIF-1 inhibitor (Cat. No. BAY 87-2243) were obtained from MedChemExpress. The LOX (bs-106158P) recombinant proteins were obtained from Bioss Bioscience Inc. The LOXL2 (11664-H08S) recombinant proteins were obtained from Sino Biological.

### 2.2. Cell culture and treatments

Primary human renal glomerular endothelial cells were obtained from Shanghai Zhongqiao Xinzhou Biotechnology (Cat. No. PRI-H-00038) demonstrated >90% purity via CD31 immunofluorescence and were confirmed pathogen-free (HIV-1/HBV/HCV negative with mycoplasma clearance); primary mouse GECs from Pronocell (Cat. No. CP-M063) were isolated through mechanical glomerular dissociation and collagenase digestion; and mouse mesangial cell line (Shanghai Zhongqiao Xinzhou, Cat. No. ZQ0194) is immortalized. Distinct from reabsorption-specialized renal epithelial cells, glomerular endothelial cells drive filtration while mesangial cells utilize gap junction-mediated syncytial coordination [28,29]. Human renal glomerular endothelial cells and mouse glomerular endothelial cells were maintained in endothelial cell culture medium (Shanghai Zhong Qiao Xin Zhou Biotechnology Co., Ltd., Cat. No. 1001) and mouse glomerular endothelial cell medium (Pronocell, Cat. No. CM-M063), respectively. In culture, they exhibit a characteristic round-to-polygonal morphology with rich cytoplasm, round/oval nuclei, and grow as confluent monolayers. Mouse glomerular mesangial cells were cultured in DMEM supplemented with 10% fetal bovine serum and 1% penicillin–streptomycin at 37°C in a humidified 5% CO₂ atmosphere. For experimental treatments, endothelial cells plated at a density of 2 × 10^6^ cells/mL in 6-well plates exposed to high glucose (30 mM) for 24 hours, followed by incubation with one of the following compounds for an additional 24 h: BAPN (0.4 mM or 40μM), PAT-1251 (10 μM), BAY 87-2243 (10 nM), or Verbascoside (10, 100 or 200 μM). After treatment, supernatants were collected and clarified by centrifugation (1000×g, 10 min, 4°C) to remove debris. Clarified supernatants were then aliquoted and stored at −80°C with protease inhibitors for subsequent analysis. All procedures were performed under sterile conditions.

### 2.3. Cell viability assay

For the mesangial cell proliferation assay induced by conditioned medium, mouse glomerular mesangial cells were seeded at a density of 1 × 10^6^ cells/mL in 96-well plates. Cells were exposed to high glucose-treated endothelial condition medium for 24 hours. Viability was subsequently quantified using a Cell Counting Kit-8 (CCK8, Abbkine, Cat. No. BMU106, China). Absorbance readings were obtained at 450 nm with a microplate reader (Thermo Scientific, USA).

### 2.4. Measurement of oxidative stress markers

Quantification of protein carbonyls and malondialdehyde (MDA) levels was performed utilizing commercially sourced assay kits, specifically obtained from Nanjing Jiancheng Bioengineering Institute (Nanjing, China) and Biyuntian (BBI Life Sciences, Shanghai, China).

### 2.5. Diabetic nephropathy modeling

Male db/m (C57BLKS/J) and db/db mice were obtained from Hangzhou Ziyuan Biotechnology Co., Ltd. The mice were housed under specific pathogen-free (SPF) conditions with a controlled temperature (22 ± 2°C), humidity (56 ± 5%), and a 12-hour light/dark cycle, with ad libitum access to food and water. At 6 weeks of age, db/db mice were maintained on a high-protein diet for an additional 6 weeks. Urinary microalbumin-to-creatinine ratio (mAlb/Cr) was measured. mAlb/Cr levels higher than those in db/m mice confirmed successful DKD model establishment. For the Verbascoside treatment, mice were randomly (Randomized by random number table) divided into Control group, Model group, and Verbascoside group (70 mg/kg/day); dosages referenced to prior studies [30,31]. The sample size was selected based on previous research [32,33]. The Verbascoside treatment was administered by gastric lavage for 6 weeks. The Verbascoside was dissolved in 0.5% (w/v) CMC-Na. The control group in our experiments is db/m mice received an equivalent volume of 0.5% (w/v) CMC-Na. The study did not have humane endpoints. At the experimental endpoint, mice were euthanized via cervical dislocation for tissue collection. Health status was monitored twice daily. The research protocols were approved by the Ethics Commission of Animal Care of the Hebei Yiling Chinese Medicine Research Institute, and experiments were conducted in accordance with the approved guidelines (approval number: N2023111).

### 2.6. Urine collection and albuminuria assessment

Twenty-four-hour specimens were gathered from mice housed in metabolic cages. Measurements of urinary albumin concentration were conducted utilizing a commercial mouse albumin ELISA kit (Abcam, ab108792), while creatinine levels were determined with a specific assay kit (Sigma, MAK080). The degree of albuminuria was assessed by calculating the albumin-to-creatinine ratio (ACR), expressed as micrograms per gram (μg/g).

### 2.7. Renal histopathology

Kidney specimens underwent fixation in 4% paraformaldehyde prior to paraffin embedding and thin-sectioning at 4 µm. Subsequent sections were processed through routine hematoxylin–eosin (H&E) and periodic acid-Schiff (PAS) staining protocols. Histopathological evaluation was performed through blinded assessment by two renal pathologists, who independently scored all kidney sections per experimental group.

### 2.8. Quantitative PCR

Total RNA extraction from both renal tissues and cultured cells was carried out using TRIzol reagent (TransGen Biotech, Beijing, China; Cat ER501-01) in accordance with the manufacturer's instructions. Briefly, after lysis, RNA was separated by chloroform phase separation, precipitated with isopropanol, washed with 75% ethanol, and finally dissolved in DEPC-treated water. RNA purity was assessed spectrophotometrically by measuring A260/A280 absorbance ratios with a NanoDrop instrument (Thermo Fisher Scientific). Complementary DNA (cDNA) synthesis from the RNA template subsequently employed a reverse transcription kit (Promega, Madison, USA, A5001). Using the resulting cDNA as template and GAPDH primers for internal control, polymerase chain reaction (PCR) amplification was performed. Quantitative gene expression analysis applied the 2-ΔΔCt method. The specific primer sequences utilized are detailed in Supplementary Table 1.

### 2.9. Immunofluorescence (IF) staining

Following embedding in Sakura Tissue-Tek OCT Compound (Sakura, USA, 4583), kidney tissues were sectioned at 5 µm thickness. These sections underwent fixation in 4% paraformaldehyde (10 minutes) and blocking with 5% BSA (30 minutes). Primary antibody incubation was performed overnight at 4°C using the following: LOX antibody (abcam, ab-174316, 1:100), LOXL2 antibody (leading biology, LBIRA1098, 1:100), CD31 antibody (Santa Cruz, sc-18916, 1:250). Sections were subsequently treated for 40 minutes in the dark with Alexa Fluor-conjugated secondary antibodies corresponding to primary antibodies: Abcam ab150077 (1:500), abcam ab150160 (1:500). DAPI nuclear counterstaining (SouthernBiotech, 0100-20) preceded imaging. High-resolution confocal microscopy utilized a Zeiss LSM710 system (Germany), with subsequent image analysis performed in ImageJ.

Mouse glomerular mesangial cells were seeded at a density of 1 × 10^6^ cells/mL in 35 mm glass-bottom dishes for immunofluorescence staining. Cultured glomerular mesangial cells were fixed in 4% paraformaldehyde (10 minutes, RT), blocked with 5% BSA (30 minutes), and permeabilized using 0.2% (v/v) Triton X-100 (Solarbio, China, T8220, 60 min). Cells were exposed overnight at 4°C to Ki67 primary antibody (Affinity AF0198 1:250) and Collagen IV antibody (Abcam, ab6586 1:250), followed by a 1-hour incubation combining Alexa Fluor594-conjugated secondary antibody (Abcam, ab150077, 1:500) with CoraLite® Plus 488-Phalloidin (Proteintech, PF00001). Mounting employed DAPI-containing medium. Images were acquired using the Zeiss LSM710 confocal microscope and analyzed with ImageJ.

### 2.10. siRNA transfection

Human renal glomerular endothelial cells were seeded at a density of 1 × 10^6^ cells/mL into 35 mm dishes and transfected with 50 nM HIF1α siRNA (Beijing OriGene Technology Co., Ltd., Cat. No. HP205393, Supplementary Table 1) in Gibco Opti-MEM reduced serum medium using Lipofectamine RNAiMAX (Invitrogen, 13778500, USA). A scrambled siRNA with no homology to rat genes was used as negative control. HIF1α mRNA and protein expression were assessed at 24 and 48 h post-transfection to confirm knockdown efficiency.

### 2.11. Western blot

To detect the knockdown efficiency of HIF1α, proteins were extracted with RIPA lysis buffer (Seven Biotechnology, SW104-01) and quantified using a BCA assay kit (Beyotime, P0012). After denaturation as per manufacturer's protocol, samples were electrophoresed on 4-20% gradient gels (GenScript, M00657) and transferred onto nitrocellulose membranes (Pall, 66485) using a semidry transfer system (GenScript). Membranes were blocked with LI-COR blocking buffer (927-60001) for 1 hour at room temperature, then incubated overnight at 4°C or 2 hours at 37°C with primary antibodies: anti-HIF1α (Cell Signaling Technology, Inc., Cat. No.36169s, 1:1000) and anti-GAPDH (Proteintech, Cat. No.60004-1-Ig, 1:50000). Following TBST washes, membranes were incubated for 1 hour at room temperature with secondary antibodies: anti-rabbit IgG (Abcam, ab175773, 1:10,000) or anti-mouse IgG (Abcam, ab175737, 1:10,000). Protein bands were visualized using an Odyssey CLX imaging system (LI-COR) and quantified with ImageJ software, with expression levels normalized to GAPDH.

### 2.12. Transcriptome analysis

Total RNA was isolated from samples using TRIzol reagent. Isolated RNA underwent rigorous quality assessment, confirming sufficient quantity (>50 ng/μL concentration) and integrity (RIN >7.0), with total mass exceeding 1 µg per sample. Polyadenylated mRNA was selectively purified with oligo (dT) magnetic beads followed by thermal fragmentation at 94°C for 5–7 minutes. First-strand complementary DNA synthesis from fragmented RNA utilized SuperScript™ II Reverse Transcriptase. Subsequent double-stranded DNA (dsDNA) synthesis employed E. coli DNA polymerase I and RNase H, incorporating dUTP to generate blunt-ended products. Size selection (300 bp ± 50 bp target) and purification occurred via magnetic bead separation. Strand specificity was introduced through UDG-mediated excision of dU-containing strands. Finally, PCR amplification generated strand-specific libraries suitable for paired-end sequencing (PE150 configuration), which was performed on the Illumina NovaSeq™ 6000 platform.

### 2.13. Proteomics analysis

For proteomic analysis, human renal glomerular endothelial cells were plated at a density of 2 × 10^6^ cells/mL and cultured in high-glucose medium (30 mmol/L) for 24 hours. The medium was then replaced with serum-free medium for 6 hours. After incubation, the conditioned medium was collected and centrifuged at 300×g for 10 minutes to obtain the supernatant. Samples were concentrated using ultrafiltration tubes. Initial centrifugation at 5000 g was performed until reaching 500 μL remaining on the filter membrane, followed by filtrate recovery. Buffer exchange was implemented by adding 5 mL PBS to the filter membrane and centrifuging at 5000 g until 350 μL remained. This washing procedure was repeated until the filtrate showed no visible color. From the concentrated sample, 100 μL aliquots underwent lysis through addition of 1% sodium deoxycholate lysate and 1X Roche Cocktail with thorough vortex mixing. Protein quantification proceeded via the BCA method, with subsequent analysis of selected samples by SDS-PAGE electrophoresis. The remaining protein solutions were alkylated: DTT was added to 10 mM final concentration and incubated at 37°C for 1 hour, followed by addition of IAA to 20 mM final concentration and dark incubation for 30 minutes. Trypsin digestion was performed at 37°C for 14–16 hours using a 50:1 protein:enzyme ratio. Resultant peptides underwent desalting using Waters solid-phase extraction cartridges followed by vacuum drying, with storage at −20°C. For liquid chromatography-mass spectrometry, mobile phases A (0.1% formic acid in water) and B (80% acetonitrile) were prepared. Dried peptides were reconstituted in 0.1% formic acid and centrifuged (20,000 g, 10 min). Chromatographic separation employed a Thermo Scientific Vanquish Neo UHPLC system with a 150 mm ES906 column under a 2.5 μl/min flow rate using an 8 min gradient: 0–4 min (4–25% B), 4–6.9 min (25–35% B), 6.9–7.3 min (35–99% B), and 7.3–8.0 min (99% B isocratic). Eluted peptides underwent DIA-mode acquisition on a Thermo Astral mass spectrometer with these parameters: normalized collision energy 25%, default charge 2, resolution 240,000, scan cycle 0.6 s, m/z range 380–980, AGC target 500%. Fragment ion scans utilized a maximum injection time of 3 ms across 300 variable windows (2 Th width, 380–980 m/z).

### 2.14. Protein–protein interaction

To identify core pathogenic proteins, we extracted common differentially expressed genes from transcriptomic and proteomic data, submitted the obtained dataset to the STRING database (https://string-db.org/) to construct a protein-protein interaction (PPI) network with a confidence threshold of 0.7 [34],and visualized the network using Cytoscape v3.9.0 [35].

### 2.15. Molecular docking

Molecular docking analysis was conducted using AutoDock Vina 1.1.2 to assess potential binding interactions. The LOX protein structure was predicted using AlphaFold (model AF-P28300-F1-model_v4; https://alphafold.ebi.ac.uk/entry/P28300), while the LOXL2 structure (PDB ID 5ZE3) was retrieved from the RCSB Protein Data Bank (https://www.rcsb.org/). A library of 123 characterized phytochemicals was obtained as SDF files from PubChem (https://pubchem.ncbi.nlm.nih.gov/). These compound structures were converted to MOL2 format with Open Babel 2.4.1 and subsequently energy-minimized using PyRx 0.8. Prior to docking, protein structures underwent preparation consisting of water removal, hydrogen addition, and Gasteiger charge calculation, followed by conversion to PDBQT format. Docking simulations employed a grid box centered at coordinates x = 213.217, y = 214.136, z = 212.095 with dimensions 18.75 Å × 17.25 Å × 28.5 Å, using rigid receptor parameters (exhaustiveness = 8, energy range = 3). Compound-receptor interaction patterns were visualized using PyMOL 2.6.0 (http://www.pymol.org/).

### 2.16. Bio-layer interferometry (BLI) assay

The binding interactions of natural compounds with target proteins LOX and LOXL2 underwent characterization using label-free bio-layer interferometry (BLI) on an Octet Red 96 platform (ForteBio, USA). Analyses employed a detection buffer composition of 1× PBS (pH 7.4), 0.04% Tween-20, and 0.1% DMSO. Purified His-tagged LOX and LOXL2 proteins were immobilized onto nickel-charged (Ni-NTA) biosensors (ForteBio, 18-5101), implementing a sequence comprising baseline equilibration, protein coupling, and secondary equilibration steps. Immobilization continued until achieving saturated response shifts of 4–6 nm. Concentration-dependent binding kinetic assays involved exposing immobilized LOX or LOXL2 to test compounds serially diluted two-fold across the 6.25–100 μM range, with both the association and dissociation phases monitored for 90 s. Buffer-loaded wells supplied corrections for baseline drift. Reference controls with Ni-NTA sensors exposed solely to buffer provided signals for nonspecific binding subtraction. Raw kinetic data underwent processing via double-reference subtraction to account for background signals and nonspecific interactions. The binding affinity constant (K_D_) was determined by globally fitting the corrected kinetic traces to a 1:1 binding model, where K_D_ equals the dissociation rate constant (k_dis_) divided by the association rate constant (k_on_). This methodology provided a sensitive, high-throughput approach demonstrating suitability for real-time, label-free compound-protein interaction analysis.

### 2.17. LOX activity measurement

Lysyl Oxidase (LOX) Assay Kit was purchased from MERCK (Cat. No.MAK555-1KT). Briefly, treat glomerular endothelial cells in 96-well plates (1 × 10^6^ cells/mL) with varying doses of VB and LOX inhibitors. Include negative controls containing medium alone to measure background fluorescence. Aspirate the conditioned medium. Add the LOX/HRP working solution to the harvested medium. Combine 50 µL of working solution with 50 µL of cell culture medium per well. Protect the reaction mixture from light, incubate at 37°C for 10–30 minutes, and immediately monitor fluorescence development using a microplate reader at excitation/emission wavelengths of 540/590 nm. Process all samples and standards in duplicate.

### 2.18. Statistics

Data are represented as the mean ± standard deviation (SD), and all statistical analyses and graphing were performed using GraphPad Prism8.0.2 (GraphPad, SanDiego, United States). No data/animals were excluded. Data were checked for normal distribution (Shapiro–Wilk test) and homogeneity of variance (Levene's test for equality of variance) before choosing the appropriate statistical test. For comparisons between two groups, Student's t-test (two-tailed) was applied. One-way ANOVA was used to determine differences among multiple groups, and post hoc comparisons were made using Tukey's post hoc test. A *p* value lower than 0.05 was considered statistically significant.

## 3. Results

### 3.1. Transcriptomic characterization of renal pathology in diabetic nephropathy mice

**Figure 1.**
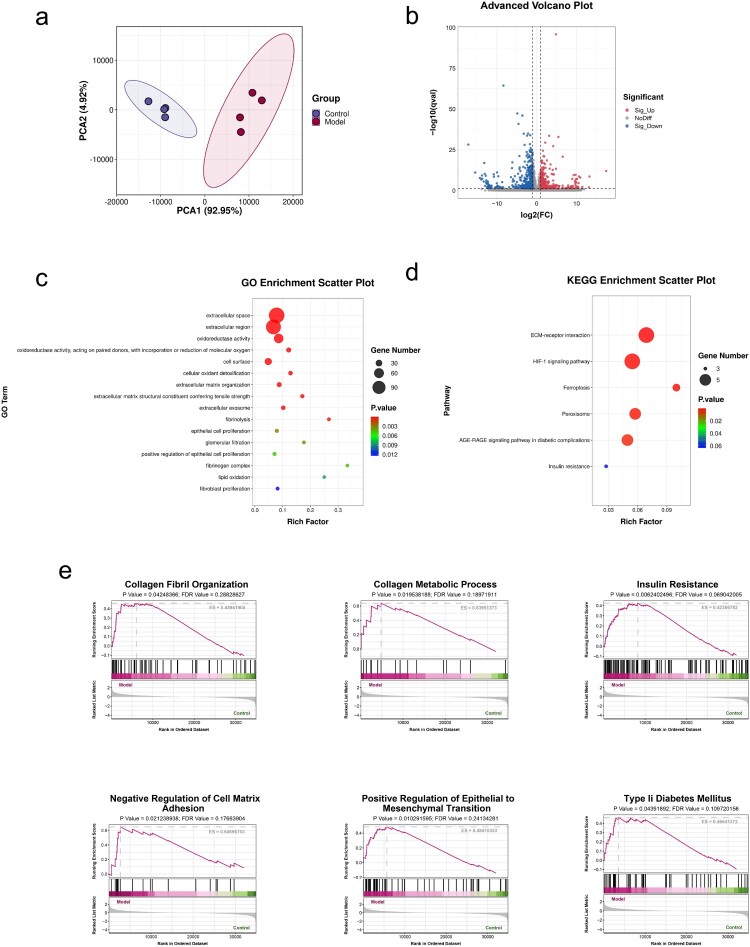
Transcriptomic profiling of diabetic nephropathy. (a) PCA of renal gene expression showing distinct separation between DN model and healthy controls, confirming diabetic nephropathy induction. (b) Volcano plot of differential gene expression in DN vs controls. (c) GO enrichment analysis of transcriptomic data. (d) Key KEGG enriched pathways of diabetic complications, oxidation regulation, and ECM-receptor interaction. (e) GSEA identifying upregulated DN pathogenesis pathways involved in EMT regulation, collagen organization, and cell adhesion dysregulation. *n* = 4.

We established DN mouse model (Supplementary Figure 1). Renal transcriptomic analysis in the DN model revealed a profound alteration of the renal gene expression landscape. Principal component analysis (PCA) exhibited a distinct and significant separation between the transcriptional patterns of the model group and the healthy control group, confirming the successful induction of the diabetic nephropathy phenotype (Figure 1(a)). Volcano plot analysis identified substantial differential gene expression in the model group compared to controls, with 410 genes significantly upregulated (53.3%) and 359 genes significantly downregulated (46.7%) (Figure 1(b)). Functional annotation using Gene Ontology (GO) and KEGG pathway enrichment analysis consistently highlighted the dysregulation of pathways directly linked to renal injury and dysfunction (Figure 1(c,d)). Key enriched pathways included glomerular filtration (GO:0003094), fibroblast proliferation (GO:0048144), fibrinolysis (GO:0042730), fibrinogen complex (GO:0005577), epithelial cell proliferation (GO:0050673/GO:0050679), insulin resistance (mmu04931), and the AGE-RAGE signaling pathway in diabetic complications (mmu04933), collectively corroborating hallmark renal pathology in our DN model.

Notably, our analysis robustly identified enrichment in multiple pathways associated with oxidative stress regulation, encompassing oxidoreductase activity (GO:0016491/GO:0016705), cellular oxidant detoxification (GO:0098869), lipid oxidation (GO:0034440), peroxisome function (mmu04146), ferroptosis (mmu04216), and the HIF-1 signaling pathway (mmu04066) (Figure 1(c,d)). Simultaneously, pronounced enrichment was observed in pathways related to extracellular proteins (GO:0005615/GO:0005576/GO:0009986), ECM organization (GO:0030198/GO:0030020), and specifically ECM-receptor interactions (mmu04512) (Figure 1(c,d)). Gene Set Enrichment Analysis (GSEA) further validated these findings and uncovered significant upregulation in the model group of pathways intrinsic to DN pathogenesis, including insulin resistance, type II diabetes mellitus, positive regulation of epithelial to mesenchymal transition (EMT), negative regulation of cell-matrix adhesion, collagen fibril organization, and collagen metabolic process (Figure 1(e)). Taken together, these comprehensive transcriptomic results strongly indicate that dysregulation in oxidative defense systems, profound alterations in extracellular protein/ECM homeostasis and signaling, and pathological processes like EMT are fundamental contributors mediating renal injury in this murine model of diabetic nephropathy.

### 3.2. Proteomic profiling reveals oxidative stress signature and validates LOX upregulation in endothelial secretome

**Figure 2.**
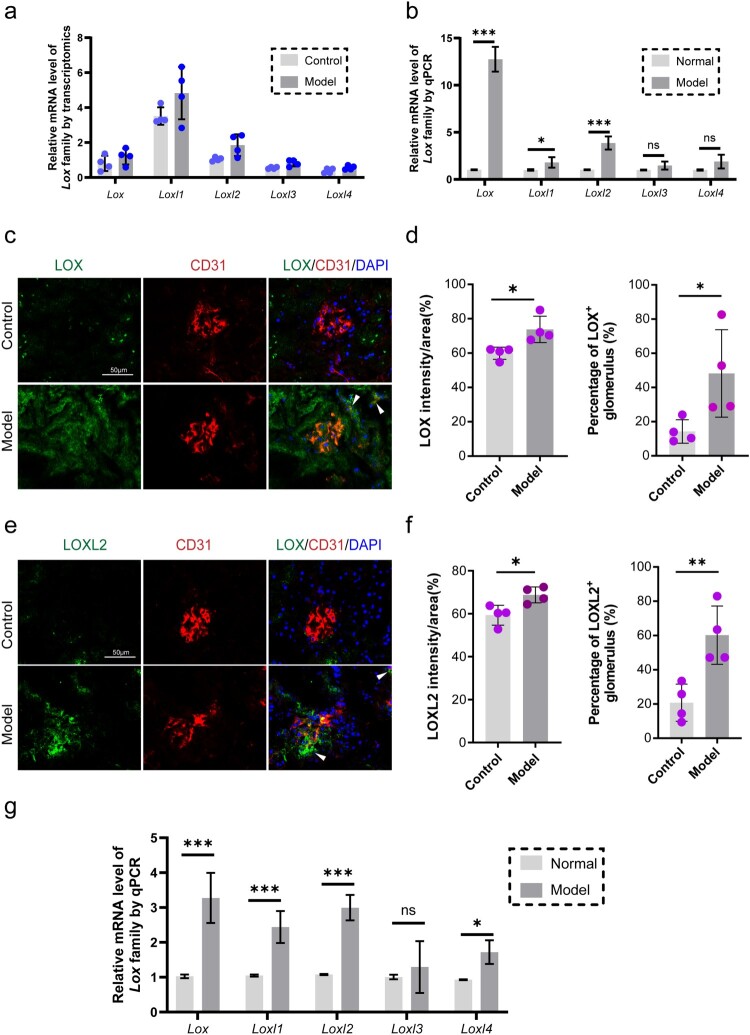
LOX family upregulation in diabetic nephropathy is endothelial-specific. (a) Transcriptomic analysis of LOX family genes (*Lox, Loxl1*, *Loxl2*, *Loxl3*, and *Loxl4*) in db/db diabetic mouse kidneys vs. controls, showing upregulation of *Lox*, *Loxl1,* and *Loxl2*. *n* = 4 (b) qPCR validation confirming increased renal mRNA levels of *Lox*, *Loxl1,* and *Loxl2* in the DN model group vs. normal controls. *n* = 5. (c–f) Immunofluorescence co-localization analysis specifying upregulation of LOX and LOXL2 protein expression within kidney endothelial cells of db/db mice compared to controls. n = 4. White arrows indicate the expressions of LOX and LOXL2 outside the glomerular endothelial cells. (g) qPCR analysis demonstrates induction of *Lox*, *Loxl1*, *Loxl2*, and *Loxl4* mRNA in mouse glomerular endothelial cells exposed to high glucose vs. normal glucose. n = 5. *p < 0.05, ***p < 0.001. Error bars indicate SD.

Building upon our transcriptomic findings highlighting oxidative stress and extracellular matrix (ECM) dysregulation as central to DN pathogenesis, we prioritized the lysyl oxidase (LOX) family as potential key mediators, given their established roles in ECM crosslinking and response to oxidative stress. Transcriptomic analysis revealed significant upregulation of multiple LOX family members in db/db mouse kidneys compared to controls, with *Lox*, *Loxl1* and *Loxl2* exhibiting the most pronounced increases (Figure 2(a)). Consistent with the RNA-seq data, quantitative real-time PCR (qPCR) analysis confirmed a substantial elevation in renal mRNA levels of *Lox*, *Loxl1* and *Loxl2* in the diabetic nephropathy model group relative to normal controls (Figure 2(b)).

To identify the cellular source responsible for elevated LOX family expression in DN, we performed immunofluorescence co-localization analyses on kidney sections. Specific upregulation of LOX and LOXL2 protein expression was observed within endothelial cells of db/db mice compared to controls (Figure 2(c–f)). To further investigate this endothelial-specific response to hyperglycemia, we cultured mouse glomerular endothelial cells (mGECs) and subjected them to high glucose (HG, 30 mM) conditions. qPCR analysis demonstrated that HG treatment significantly induced the mRNA expression of *Lox*, *Loxl1*, *Loxl2*, and *Loxl4* in these endothelial cells (Figure 2(g)). Collectively, these results provide converging evidence from tissue and cellular models that endothelial-derived extracellular LOX family proteins are markedly upregulated in diabetic nephropathy, suggesting a potential pathogenic role in disease progression.

**Figure 3.**
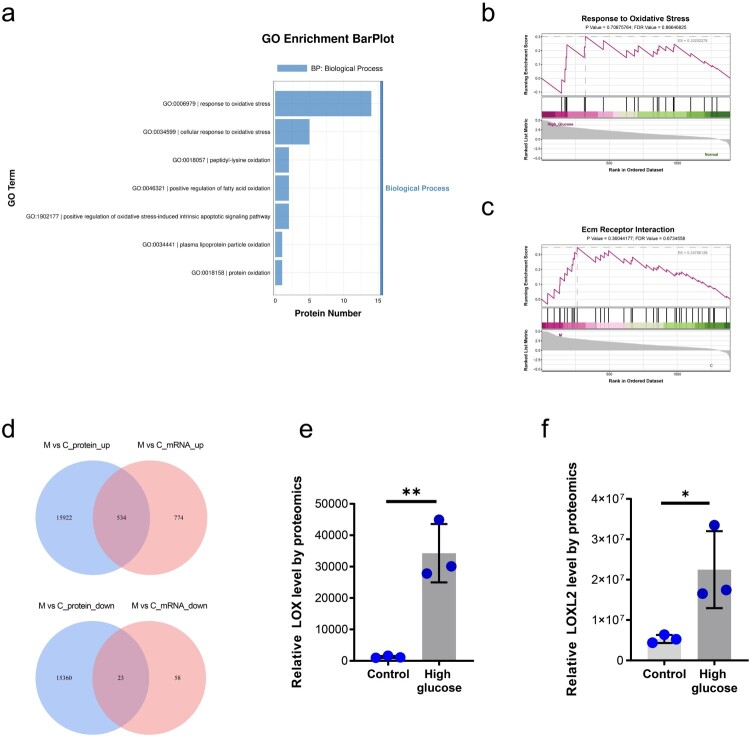
Proteomic profiling of the glomerular endothelial cell secretome under high glucose. (a) GO enrichment analysis of conditioned media from high glucose-treated GECs showing significant enrichment in pathways related to oxidative stress, lipid peroxidation, and protein oxidation. (b,c) GSEA confirming significant upregulation of oxidative stress and ECM-receptor interaction signaling pathways in HG-treated GECs. (d) Cross-referencing transcriptomic and proteomic datasets identifying commonly upregulated and commonly downregulated genes/proteins in diabetic kidneys and HG-EC CM. (e,f) Significant elevation of secreted LOX and LOXL2 proteins in HG-EC CM vs. control CM. *n* = 3; *p < 0.05, **p < 0.01. Error bars indicate SD.

To further investigate endothelial responses under diabetic conditions, we performed proteomic sequencing on conditioned media (CM) from glomerular endothelial cells (GECs) cultured under high glucose (HG, 30 mM). Analysis of the HG-EC CM proteome revealed significant enrichment for pathways associated with oxidative stress (e.g. GO:0006979 Response to oxidative stress, GO:0034599 Cellular response to oxidative stress) and lipid peroxidation (e.g. GO:0010430 Fatty acid omega oxidation, GO:0046321 Very long-chain fatty acid metabolic process) (Figure 3(a)). We also observed enrichment in protein oxidation processes (e.g. GO:0018057 Peptidyl-lysine oxidation, GO:0034441 Plasma lipoprotein particle oxidation, GO:0018158 Protein oxidation) (Figure 3(a)). Furthermore, Gene Set Enrichment Analysis (GSEA) demonstrated significant upregulation of the oxidative stress and ECM-receptor interaction signaling in HG-treated GECs (Figure 3(b,c)). These findings support the concept that GECs subjected to hyperglycemia are a significant source and effector of oxidative stress, potentially mediating ECM remodeling during DN pathogenesis. To identify core mediators linking endothelial oxidative stress to ECM dysregulation, we cross-referenced significantly altered transcripts from the diabetic kidney transcriptome with significantly altered proteins identified in the HG-EC CM proteome. This integrated analysis identified 534 commonly upregulated genes/proteins and 23 commonly downregulated genes/proteins (Figure 3(d)). We performed PPI network analysis on these co-regulated proteins and validated the mRNA upregulation of THBS1, CCL2, and SERPINE1 as hub interactors with high connectivity (Supplementary Figure 2a, b). Notably, LOX and LOXL2 proteins were also significantly elevated within the HG-EC CM, exhibiting fold increases of 27-fold and 4.2-fold, respectively, compared to control CM (Figure 3(e,f)). This robust upregulation of secreted LOX proteins in the endothelial secretome directly validates our initial transcriptomic and cellular localization findings, strongly implicating LOX family members as key factors in endothelial-mediated oxidative stress and pathological ECM remodeling in DN.

### 3.3. Endothelial LOX/LOXL2 inhibition protects mesangial cells from high glucose-induced oxidative stress and fibrosis *in vitro*

**Figure 4.**
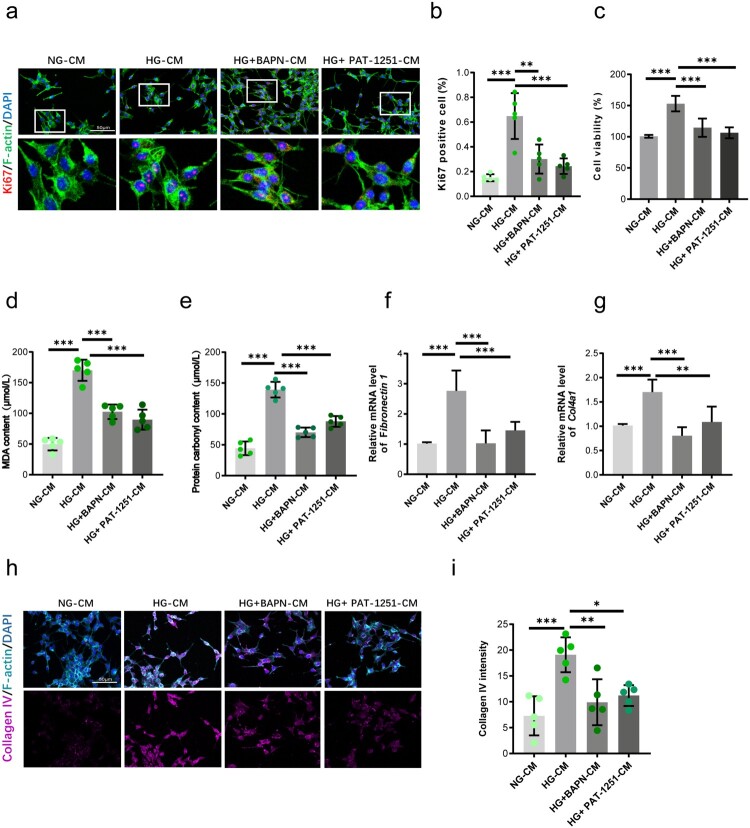
Inhibition of endothelial LOX/LOXL2 protects mesangial cells from high glucose-induced damage. (a–c) Suppression of mesangial cell proliferation and viability by conditioned media from HG-exposed GEnCs treated with BAPN (HG + BAPN-CM) or PAT-1251 (HG + PAT-1251-CM), compared to damaging HG-CM. *n* = 5. (d,e) Attenuation of HG-CM-induced lipid peroxidation and protein carbonylation in mesangial cells by endothelial LOX/LOXL2 inhibition. *n* = 5. (f,g) Suppression of fibrotic gene expression (FN1, Col4a1) in mesangial cells exposed to inhibitor-treated CM. *n* = 5. (h,i) Reduced collagen deposition in mesangial cells treated with BAPN-CM or PAT-1251-CM vs. HG-CM. n = 5. *p < 0.05, **p < 0.01, ***p < 0.001. Error bars indicate SD.

Building on the pathogenic role of endothelial LOX family proteins, we hypothesized that inhibiting specific LOX/LOXL members secreted by endothelial cells would attenuate functional and pathological changes in mesangial cells. Using *in vitro* modeling, we first established safe and effective doses of inhibitors for pan Lysyl Oxidase (BAPN) and LOXL2 (PAT-1251). Previous reports and our CCK8 assays identified 0.4 mM BAPN and 10 μM PAT-1251 as non-toxic concentrations (Supplementary Figure 3) [36,37]. To assess endothelial-to-mesangial paracrine signaling, we collected conditioned medium (CM) from four groups: GEnCs under normal glucose (NG-CM), GEnCs under high glucose (HG-CM), HG-GEnCs treated with the LOX inhibitor BAPN (HG + BAPN-CM), and HG-GEnCs treated with the LOXL2 inhibitor PAT-1251 (HG + PAT-1251-CM). This CM was then applied to mesangial cells. Mesangial cells exposed to HG-CM exhibited significantly higher proliferation and viability compared to those treated with NG-CM (Figure 4(a–c)). Strikingly, CM from HG-GEnCs treated with either BAPN or PAT-1251 significantly supressed mesangial cell proliferation and vitality (Figure 4(a–c)). Further analysis revealed that HG-CM induced prominent oxidative stress in mesangial cells. This was evidenced by significant increases in lipid peroxidation (measured by MDA content) and protein carbonylation (Figure 4(d,e)). Treatment of endothelial cells with BAPN or PAT-1251 during high glucose exposure markedly reduced these oxidative markers in the subsequent mesangial cells (Figure 4(d,e)). Critically, exposure to HG-CM drove mesangial cells towards a pro-fibrotic phenotype. qPCR analysis showed significant upregulation of key fibrosis markers, including fibronectin (*FN1*), and collagen type IV alpha 1 (*Col4a1*) mRNA (Figure 4(f,g)). Supporting this, immunostaining confirmed increased collagen deposition in mesangial cells treated with HG-CM compared to NG-CM (Figure 4(h,i)). Notably, CM from HG-GEnCs treated with either LOX inhibitor (BAPN-CM) or LOXL2 inhibitor (PAT-1251-CM) significantly attenuated both the mRNA upregulation of pro-fibrotic genes (Figure 4(h,i)) and the increase in collagen deposition (Figure 4(h,i)). Collectively, these results demonstrate that specific inhibition of endothelial LOX or LOXL2 activity interrupts deleterious paracrine signaling under high glucose conditions. This interruption protects mesangial cells by attenuating the detrimental effects on proliferation and viability, reducing lipid and protein oxidation, and suppressing the expression of fibrotic mediators and collagen accumulation. This establishes a critical pathogenic role for endothelial-derived LOX and LOXL2 in promoting mesangial cell injury, oxidative stress, and fibrosis, key features of diabetic nephropathy.

### 3.4. HIF-1α inhibition attenuates mesangial proliferation by suppressing endothelial LOX/LOXL2 signaling

**Figure 5.**
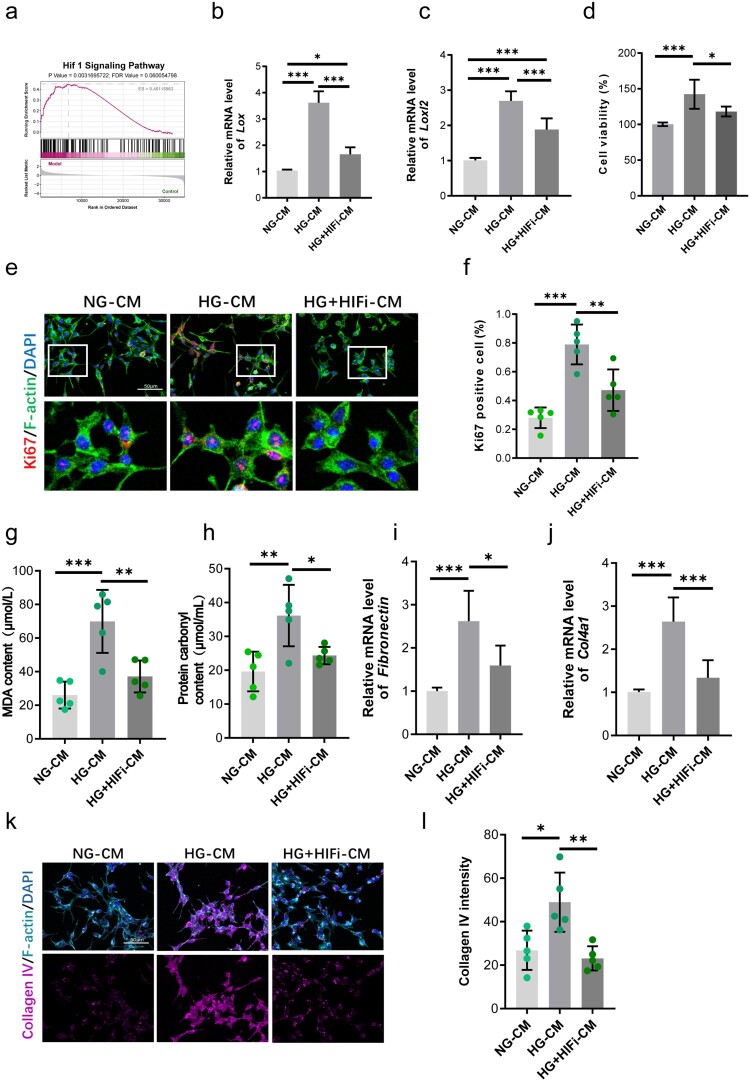
HIF-1α drives endothelial LOX/LOXL2-mediated mesangial injury. (a) Transcriptional upregulation of HIF-1α pathways in diabetic kidneys. *n* = 4. (b, c) Suppression of LOX and LOXL2 mRNA in HG-exposed GECs by HIF-1α inhibition. *n* = 5. (d-f) Suppression of mesangial proliferation/viability deficits by CM from HIF-inhibited GEnCs (HG + HIFi-CM). n = 5. (g, h) Reduction of oxidative stress markers in mesangial cells treated with HG + HIFi-CM. n = 5. (i-l) Attenuation of fibrotic gene expression (FN1, Col4a1) and collagen deposition in mesangial cells receiving HG + HIFi-CM. *n* = 5. *p < 0.05, **p < 0.01, ***p < 0.001. Error bars indicate SD.

Analysis of transcriptional changes associated with high glucose exposure highlighted significant upregulation of HIF-1α pathways in DN mice kidneys (Figure 5(a)), consistent with this known master regulator's role in diabetic complications and its direct transcriptional control of LOX and LOXL2 expression [38, 39]. To determine if HIF-1α drives downstream pathogenic LOX signaling during intercellular crosstalk, we investigated the effects of HIF-1α inhibition using BAY 87-2243. Following previous report and determination of a safe concentration at 10 nM (Supplementary Figure 4a) [40], we confirm that inhibition of HIF-1α decreased the mRNA levels of endothelial *Lox* and *Loxl2* (Figure 5(b,c)). We also performed siRNA-mediated knockdown of HIF-1α (with knockdown efficiency confirmed at 50% by WB, Supplementary Figure 4b), which led to significant reductions in LOX and LOXL2 mRNA expression by around 67% and 72%, respectively (Supplementary Figure 4(c,d)). We then generated conditioned media (CM) from GEnCs under three conditions: normal glucose (NG-CM), high glucose (HG-CM), and high glucose with HIF-1α inhibitor (HG + HIFi-CM). Applying the CM to mesangial cells demonstrated that inhibiting HIF-1α in endothelial cells recapitulated the effects observed with direct LOX inhibition. Elevated mesangial cell proliferation and viability caused by HG-CM were significantly reduced by HG + HIFi-CM (Figure 5(d–f)). Furthermore, the pronounced HG-CM-induced oxidative stress was markedly mitigated when mesangial cells were cultured with CM from HIF-1α inhibited GEnCs (Figure 5(g,h)).

Critically, the pro-fibrotic transformation of mesangial cells triggered by HG-CM was similarly counteracted by endothelial HIF-1α blockade. Mesangial cells receiving HG-CM exhibited robust upregulation of key fibrotic genes (*FN1* and *Col4a1*) and substantial increases in collagen deposition (Figure 5(i–l)). In parallel, HG + HIFi-CM significantly suppressed aberrant fibrotic marker expression and prevented the pathological accumulation of extracellular collagen matrix in recipient mesangial cells (Figure 5(i–l)). The virtually identical capacity of HIF-1αpharmacologic inhibition to abrogate mesangial dysfunction, oxidative injury, and fibrotic activation establishes HIF-1α as the critical upstream transcriptional activator driving endothelial LOX production and mediating subsequent paracrine injury to mesangial cells in a high glucose milieu.

### 3.5. Natural compound screening identifies verbascoside as a novel LXO/LOXL2 dual inhibitor

**Figure 6.**
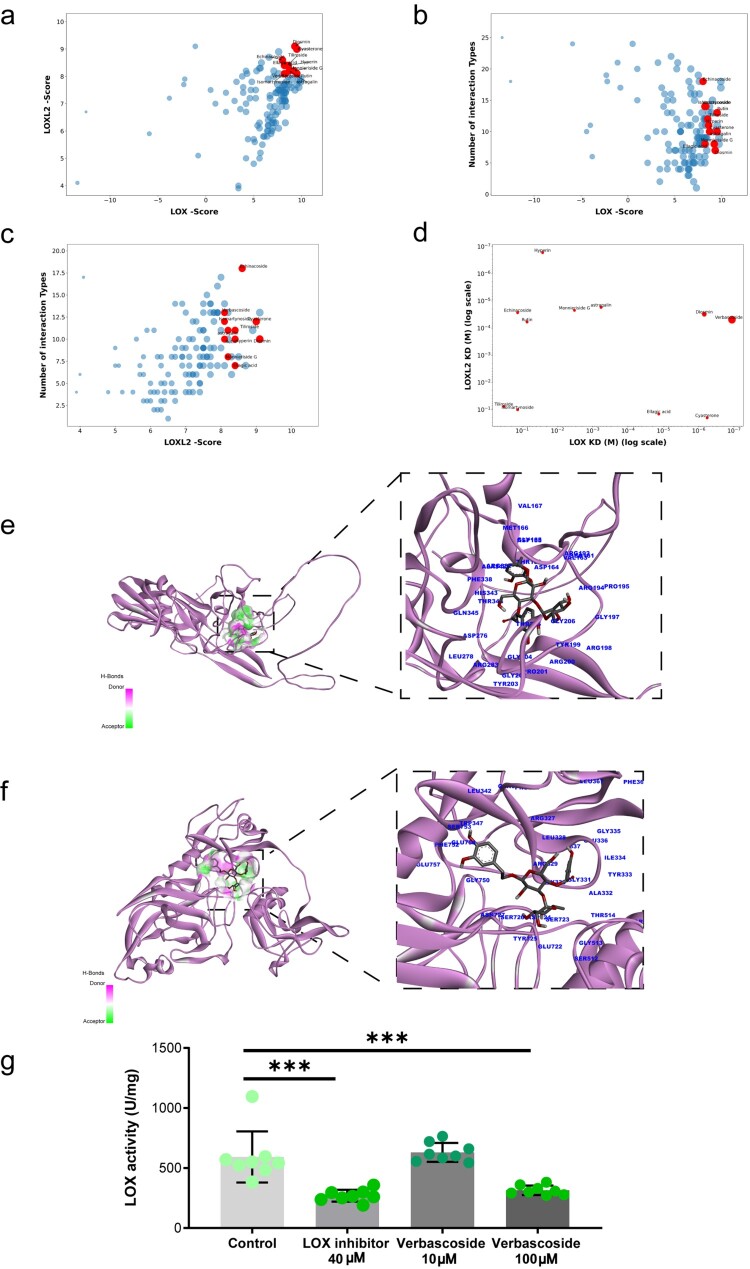
Identification of Verbascoside as a dual LOX/LOXL2 inhibitor. (a-c) Virtual screening of a natural product library against LOX/LOXL2, prioritizing 11 candidates. (d) BLI kinetics revealing Verbascoside's high affinity for LOX (K_D_ = 1.13 × 10^−^^7^M) and LOXL2 (K_D_ = 4.92 × 10^−^^5^M). (e) Molecular docking interactions of Verbascoside and LOX at LOX binding site. (f) Analogous binding interactions at LOXL2 site. (g) Verbascoside inhibits LOX enzyme activity. *n* = 8. ***p < 0.001. Error bars indicate SD.

Given the therapeutic potential of LOX/LOXL2 inhibitors in diabetic nephropathy, we conducted structure-based virtual screening of a natural product library (123 compounds derived from renal-protective botanical medicines) against LOX/LOXL2 using molecular docking to prioritize high-affinity candidates (Figure 6(a)). Using docking score (based on AutoDock Vina criteria, where < −5 indicates strong affinity) and the diversity of bonding interactions as selection criteria (Figure 6(a–c)), we prioritized eleven compounds for experimental validation: Cyasterone, Rutin, Diosmin, Monnieriside G, astragalin, Hyperin, Tiliroside, Verbascoside, Ellagie acid, Isomartynoside, and Bcbinaooside.

We then assessed binding kinetics against LOX and LOXL2 using biolayer interferometry (BLI) (Figure 6(d)). Nine of the eleven tested compounds exhibited dissociation constants (KD) ranging from 3.26 × 10^−^^1^ M to 1.13 × 10^−^^7^ M for LOX, and from 2.01 × 10^−^^1^ M to 1.68 × 10^−^^7^ M for LOXL2 (Figure 6(d)). Verbascoside (VB) – a bioactive phenylethanoid glycoside from *Cistanche deserticola* – exhibited a KD of 1.13 × 10^−^^7^ M with LOX (corresponding to a virtual docking score of −8.3) and a KD of 4.92 × 10^−^^5^ M with LOXL2 (docking score: −8.1) (Figure 6(e,f)). Molecular docking confirmed that at the LOX binding site, Verbascoside established interactions including: fourteen van der Waals bonds (Arg198, Arg192, Thr344, Phe338, Gly202, Gly206, Thr205, Asp206, Ser161, Gly197, Pro195, His343, Thr189, Asp188), five Hydrogen Bonds (Arg337, Gly204, Arg200, Asp164, Val163, Ala342), one Pi-cation bond (Met166) and one Pi-Sigma interaction (Tyr199) (Figure 6(e)). Similarly, at the LOXL2 binding site, it formed interactions with the residues: seventeen van der Waals bonds(Gly331, Gly330, Gly337, Tyr333, Leu328, Gly513, Thr514, Tyr725, Ser726, Arg478, Glu757, Gly750, Glu754, Phe752, Leu342, Ser753, Pro324), seven hydrogen bonds(Glu336, Gly335, Asp724, Ser723, Gln323, Trp347, Arg329), one Pi-cation bond(Arg327), and one Pi-alkyl interaction(Ile334) (Figure 6(f)).

To further elucidate the regulatory mechanism of VB, we examined its effect on regulating HIF1α, LOX, and LOXL2 expression, as well as LOX enzymatic activity. In consistent with previous report, Verbascoside exhibited no cytotoxicity at 200 µM (Supplementary Figure 5a) [41]. Quantitative PCR analysis revealed that VB treatment dose-dependently reduces the mRNA levels of HIF1α, LOX, and LOXL2 (Supplementary Figure 5b-d). Mover, *in vitro* enzyme activity assays demonstrated that 100 µM of VB inhibited LOX enzymatic activity (Figure 6(g)). This dual inhibition of VB on the HIF/LOX signaling pathway at both transcriptional and enzymatic levels indicates that it is a very promising compound.

### 3.6. Verbascoside ameliorates diabetic nephropathy progression

**Figure 7.**
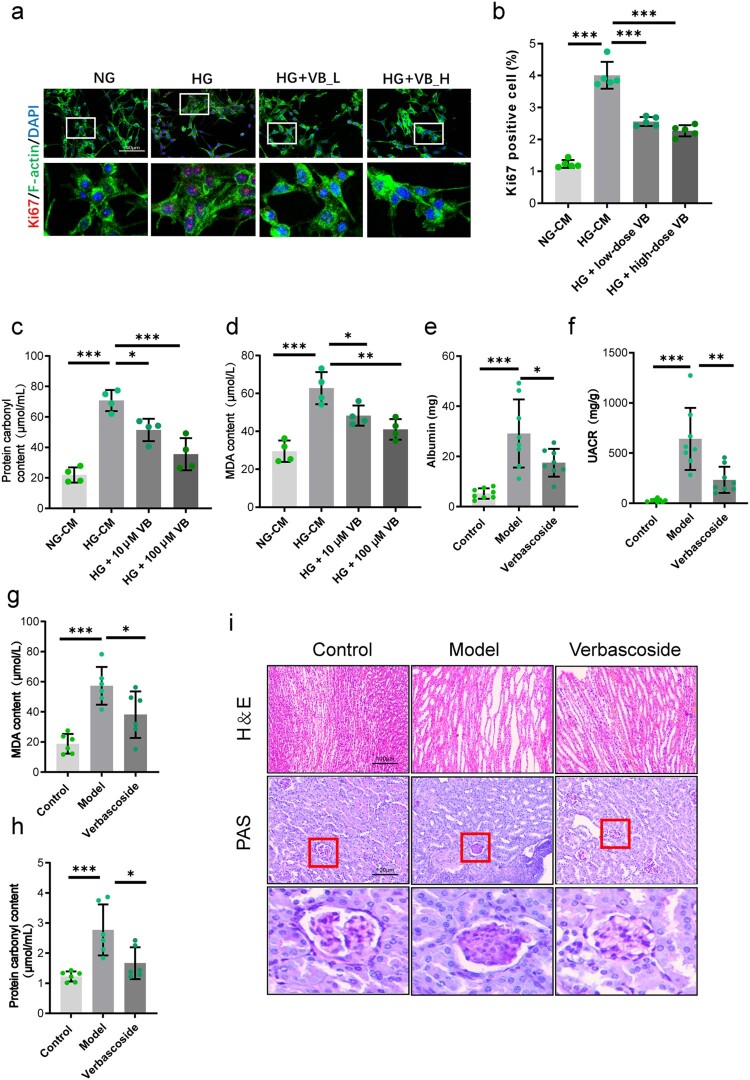
Therapeutic efficacy of Verbascoside in diabetic nephropathy. (a,b) Dose-dependent suppression of mesangial proliferation by CM from VB-treated HG-GEnCs. n = 5. (c,d) Reduction of oxidative stress markers in mesangial cells with VB-CM treatment. n = 4. (e,f) Improvement in renal function diabetic mice treated with VB. *n* = 8. (g,h) Amelioration of renal oxidative damage with VB treatment. *n* = 6. (i) Amelioration of histopathology with VB treatment. *n* = 4. *p < 0.05, **p < 0.01, ***p < 0.001. Error bars indicate SD.

To evaluate the therapeutic potential of Verbascoside in diabetic nephropathy, we first assessed its cytotoxicity on glomerular endothelial cells (GECs). Subsequent experiments with conditioned media were conducted as follows: GECs were cultured under normal glucose (NG), high glucose (HG), HG + low-dose VB (100 µM), or HG + high-dose VB (200 µM). Collected CM was then applied to mesangial cells. Results demonstrated that VB-CM dose-dependently inhibited mesangial cell proliferation (Figure 7(a,b)). VB-CM also significantly attenuated oxidative stress in mesangial cells, evidenced by reduced malondialdehyde (MDA) and protein carbonyl content (Figure 7(c,d)).

Based on these results, we performed *in vivo* experiments. Diabetic mice were administered Verbascoside (70 mg/kg/day) for 6 weeks (Supplementary Figure 6a). Notably, Verbascoside treatment decreased urinary protein excretion, urinary albumin-to-creatinine ratio (UACR), blood glucose, as well as kidney weight to body weight (Figure 7(e,f), and Supplementary Figure 6b–d), indicating preserved renal function. Verbascoside also reduced renal oxidative damage, reflected by lowered MDA and carbonyl protein levels (Figure 7(g,h)). Histopathological examination of kidney tissue (H&E and Masson's trichrome staining) further indicated that the model mice exhibited characteristic renal lesions including tubular dilation, urothelial hyperplasia, and glomerular mesangial proliferation, while VB treatment ameliorated kidney injury and glomerular mesangial matrix expansion (Figure 7(i)). Collectively, these results demonstrate VB’s potential in ameliorating diabetic kidney injury.

## 4. Discussion

The glomerular microenvironment in diabetic nephropathy (DN) undergoes profound pathological remodeling, driven by interconnected dysregulations in extracellular matrix (ECM) homeostasis, oxidative stress responses, and aberrant cellular crosstalk [42,43]. Samples for analyses were consistently collected as whole/half kidneys from both genotypes, though db/db kidney enlargement may alter tissue composition. Importantly, because LOX and LOXL2 are also expressed in the tubulointerstitium and this compartment dominates the cortical mass, bulk kidney mRNA sequencing necessarily reflects a mixture of transcripts, with tubulointerstitial sources potentially prevailing. Nevertheless, the upregulation of potential pathogenic factors was confirmed by glomerular endothelial secretome proteomics and functional validation under high glucose stress (Figures 2 and 3), supporting our conclusions despite potential transcriptomic sampling bias. Our comprehensive transcriptomic profiling of diabetic kidneys revealed a landscape dominated by alterations critical to DN pathogenesis, with significant enrichment in pathways governing ECM organization, collagen metabolic processes, and oxidoreductase activity (Figure 1). Proteomic analysis of glomerular endothelial cell (GEC) secretomes further delineated the endothelial contribution to this dysregulation under high glucose (HG) stress. Conditioned media (CM) from HG-treated GECs exhibited pronounced enrichment in proteins mediating oxidative damage (e.g. lipid peroxidation, protein oxidation) and ECM-receptor interactions (Figure 3(a–c)). Crucially, this multi-omics integration pinpointed the lysyl oxidase family, particularly LOX and LOXL2, as centrally upregulated mediators. LOX and LOXL2 transcripts surged in diabetic kidneys (Figure 2(a,b)) and were examined via immunofluorescence wherein LOX/LOXL2 intensity per glomerular area (outlined by CD31) was quantified (Figure 2(c–f)). Notably, LOX and CD31 signals were also weakly detected in interstitial peritubular capillaries (white arrows, Figures 2(c,e)), and LOX seems to be also widely expressed in tubular epithelial cells, suggesting its potential role in renal fibrosis and vascular remodeling. LOXL2 staining localized to some glomerular CD31-negative cells, indicating expression beyond glomerular endothelium (e.g. interstitial cells). This broader expression pattern of the LOX family within the diabetic kidney parenchyma warrants further exploration to fully delineate its pathological significance. While endothelial-derived TGF-β, VEGF, and exosomes may contribute to mesangial injury through HIF-1α-independent pathways, our secretome analysis definitively established HG-stressed GECs as primary sources of LOX/LOXL2 secretion (Figure 3(e,f)). Although this proteomic approach focused on soluble secreted factors in the supernatant and may not detect ECM-associated or cell-bound LOX/LOXL2 fractions, collectively these data identify endothelial-derived LOX/LOXL2 as pivotal drivers of glomerular ECM stiffening and oxidative damage.

Thrombospondin-1 (THBS1) is a major activator of TGF-β in fibrotic renal disease [44], C–C chemokine receptor type 2 (CCL2) mediates glomerular injury and interstitial fibrosis in focal segmental glomerulosclerosis [45], and plasminogen activator inhibitor-1 (SERPINE1) promotes fibrin deposition and fibrosis by inhibiting matrix degradation [46]. Beyond these established pathogenic mediators, the pathogenic influence of endothelial LOX/LOXL2 extends dynamically beyond autocrine effects to orchestrate deleterious paracrine signaling affecting key glomerular cell types [47,48]. Conditioned media harvested from HG-activated GECs triggered profound oxidative stress within mesangial cells, evidenced by marked elevations in hallmark biomarkers – malondialdehyde (MDA) signifying lipid peroxidation and reactive carbonyls indicating protein oxidation (Figure 4(d,e)). Concomitantly, HG-CM induced relentless mesangial cell proliferation, potentially through TGF-β signaling activation [49–51]. This was accompanied by a fibrotic phenotype characterized by upregulated expression of key mediators (*FN1* and *Col4a1*) and tangible pathological collagen deposition (Figure 4(f–i)). Strikingly, pharmacological inhibition of LOX or LOXL2 within the endothelial compartment prior to CM collection completely reversed all facets of mesangial dysfunction – reducing proliferative capacity, quenching oxidative stress markers, silencing fibrotic gene induction, and preventing collagen matrix accumulation (Figure 4(a–i)). Moreover, our functional interrogation and previous reports demonstrated that HIF-1α serves as the master transcriptional regulator of LOX/LOXL2 in endothelial cells exposed to hyperglycemia (Figure 5(b,c)) [39,52,53]. This compelling rescue with previous reports together underscores HIF-1α induced LOX and LOXL2 secretion as non-redundant conductors of pathogenic endothelial-to-mesangial crosstalk underpinning diabetic glomerulopathy.

Building on established evidence that Verbascoside ameliorates diabetic nephropathy through anti-inflammatory and antioxidant mechanisms [25,30,54–56]. Prior studies demonstrate its capacity to reduce albuminuria and oxidative stress in animal studies [26,57–59] Given the prominence of the LOX family axis in disease pathogenesis [60,61] targeted inhibition presents a promising therapeutic strategy. Natural products, with their established safety profiles suited for chronic management, represent an ideal source for such molecules. Utilizing structure-based virtual screening against LOX and LOXL2, we identified the phenylethanoid glycoside Verbascoside as a novel dual inhibitor exhibiting strong binding affinities validated by biolayer interferometry (Figure 6). Molecular docking elucidated extensive stable interactions at the target enzyme active sites (Figure 6(e,f)). Functionally, Verbascoside efficiently intercepted the pro-injurious endothelial secretome at the source. Treating mesangial cell with Verbascoside generated GECs conditioned media (VB-CM) significantly attenuated its proliferation *in vitro* (Figure 7(a,b)), and crucially, mitigated mesangial oxidative stress, reducing MDA and protein carbonylation levels (Figure 7(c,d)). Translating this to the *in vivo* setting established the broader therapeutic relevance demostrating diabetic mice treated with Verbascoside exhibited significantly preserved renal function, marked by decreased urinary protein excretion and UACR (Figure 7(e,f)). Kidney tissues from Verbascoside-treated animals displayed reduced oxidative damage (Figure 7(g,h)), attenuation of kidney injury and mesangial matrix expansion (Figure 7(i)). It should be noted that our 6-week intervention only assessed VB's efficacy in early DN stages; future investigations extending to 12–24 weeks with longitudinal renal function monitoring are required to evaluate its capacity to halt progression to advanced fibrosis/sclerosis. Critically, our mechanistic studies demonstrate that VB simultaneously suppresses HIF-1α-mediated *LOX/LOXL2* transcription and directly inhibits LOX enzymatic activity (Supplementary Figure 5b-d and 6g), establishing a dual blockade at both genomic and functional levels. Importantly, recent work demonstrates VB also acts as a potent SGLT2 inhibitor effective at low concentrations (8–16 μM) [56]. This multi-target engagement – concomitant inhibition of HIF-1α transcriptional regulation, LOX catalytic function, and sodium-glucose transport – explains VB's significant renoprotection at low doses: by targeting pathogenic cascades at complementary nodes (transcriptional initiation, enzymatic execution, and metabolic priming), it achieves synergistic efficacy where single-mechanism agents require higher concentrations. Collectively, Verbascoside’s efficacy arises through its multi-tiered action of suppression of the HIF-1α-LOX/LOXL2 pathway within endothelial cells, normalization of glucose metabolism and endothelial paracrine secretome thereby relieving oxidative and fibrotic stress on mesangial cells, and measurable alleviation of DN manifestations.

This work establishes endothelial LOX and LOXL2 as lynchpins within the dysregulated signaling network governing glomerular injury in diabetic nephropathy. By demonstrating their HIF-1α-driven upregulation and delineating their pathogenic downstream effects via endothelial-mesangial crosstalk, we provide a mechanistic blueprint for targeted intervention. However, the documented requirement for high VB doses *in vivo* underscores the inherent limitations of VB's native form, which are primarily attributed to its suboptimal pharmacodynamic properties, and critically low bioavailability [62–65]. Verbascoside emerges as a compelling prototype natural therapeutic agent derived from the food-medicine homologous herb *Cistanche*. Notwithstanding its natural origin and established safety background [66,67], comprehensive toxicological assessments remain essential to fully elucidate potential adverse effects for chronic human administration in DN for high dosage administration. On the other hand, to overcome these pharmacodynamic challenges, future research should focus on structural optimization strategies. These may include synthesizing metabolism-resistant prodrugs to enhance stability, developing renal-targeted nanodelivery platforms for localized accumulation, or designing novel analogs with improved LOX/LOXL2 binding kinetics while preserving molecular specificity. Such targeted modifications could substantially enhance the therapeutic potential of VB-based interventions for DKD. Success in this endeavor would unlock a mechanistically grounded therapeutic avenue with significant translational potential for diabetic nephropathy progression.

## Author  contributions

Conceptualization, M.L.; methodology, M.L. and T.K.; software, B.H., and M.S.; validation, T.K., B.H., H.L., K.L., Y.L., Z.W., S.L., and Z.X.; formal analysis, T.K., K.L., and Y.L.; investigation, T.K. and B.H.; resources, M.L.; data curation, M.L. and T.K.; writing – original draft preparation, M.L.; writing – review and editing, M.L. and T.K.; visualization, T.K. and B.H.; supervision, M.L.; project administration, M.L.. All authors have read and agreed to the published version of the manuscript.

## Supplementary Material

255374048.R2_supplementary_materials.docx

## Data Availability

The data used to support the findings of this study are available from the corresponding authors upon request**.** The mass spectrometry proteomics data has been deposited to the ProteomeXchange Consortium (https://proteomecentral.proteomexchange.org) with the dataset identifier PXD066579. The sequencing data is stored in the Sequence Read Archive (SRA) with accession number PRJNA1303041.
