# Supplementary material for: Verbascoside targets endothelial HIF-1α/ Lysyl oxidase signaling to attenuate glomerular injury in diabetic nephropathy
Source: Redox Rep. 2025 Dec 8;30(1):2598110. doi: 10.1080/13510002.2025.2598110 (PMC12687904; doi:10.1080/13510002.2025.2598110)
Supplement: 255374048.R2_supplementary_materials.docx [file YRER_A_2598110_SM2400.docx]

Supplementary Table 1 Oligonucleotide sequences

| **Quantitative PCR primers** | | |
| --- | --- | --- |
| Gene | **Forward** | **Reverse** |
| *mLox* | GGAGGCTGAAGTGGATGAGG | CCAGGAAGCCAGGTTGTCTC |
| *mLoxl1* | GGCCTCAGGGAGTGAACAT | CAATCTTGCTGTGTGGGATG |
| *mLoxl2* | GGACAACAACGTGGACAAGG | CAGGAAGTCGATGCTGTTCC |
| *mLoxl3* | CTACTGCTGCTACACTGTCTGT | GACCTTCATAGGGCTTTCTAGGA |
| *mLoxl4* | GCCAACGGACAGACCAGAG | CCAGGTCAAGGCTGACTCAAA |
| *mFN1* | ATGCCAAATCTTGCGGAGAAT | TTTGCTGCGATTGGTGACATT |
| *mCol4a1* | ATGGCTTGCCTGGAGAGATAGG | TGGTTGCCCTTTGAGTCCTGGA |
| *mThbs1* | GGTAGCTGGAAATGTGGTGCGT | GCACCGATGTTCTCCGTTGTGA |
| *mCcl2* | GCTACAAGAGGATCACCAGCAG | GTCTGGACCCATTCCTTCTTGG |
| *mSerpine1* | CCTCTTCCACAAGTCTGATGGC | GCAGTTCCACAACGTCATACTCG |
| *mGapdh* | GGTGAAGGTCGGTGTGAACG | CTCGCTCCTGGAAGATGGTG |
| *hLOX* | CAGGGATGGTGAGGTGAAGA | GGAGGCAGGTGTAGCAATGA |
| *hLOXL1* | ACAGCACCTGTGACTTCGGCAA | CGGTTATGTCGATCCACTGGCA |
| *hLOXL2* | CAGCCAGGGACACCTCATAC | GGCACTGGATCTTGTAGTTGG |
| *hLOXL3* | GCACAGTCTGTGACCGCAAGTG | CTTCACTCAGGTGGATAGCACC |
| *hLOXL4* | CCAAAGACTGGACGCGATAGCT | GGCAGTTTGTGTCCTCCAGACA |
| *hHIF1A* | TATGAGCCAGAAGAACTTTTAGGC | CACCTCTTTTGGCAAGCATC |
| *hGAPDH* | GGAGCGAGATCCCTCCAAAAT | GGCTGTTGTCATACTTCTCATGG |
| **Small interfering RNA sequences‌‌** | | |
| Gene | **Forward** | **Reverse** |
| *hHIF1A* | CAGAAAUGGCCUUGUGAAATT | UUUCACAAGGCCAUUUCUGTT |
| Control | UUCUCCGAACGUGUCACGUTT | ACGUGACACGUUCGGAGAATT |

**Quantitative PCR primers and Small interfering RNA sequences of the experiment.**


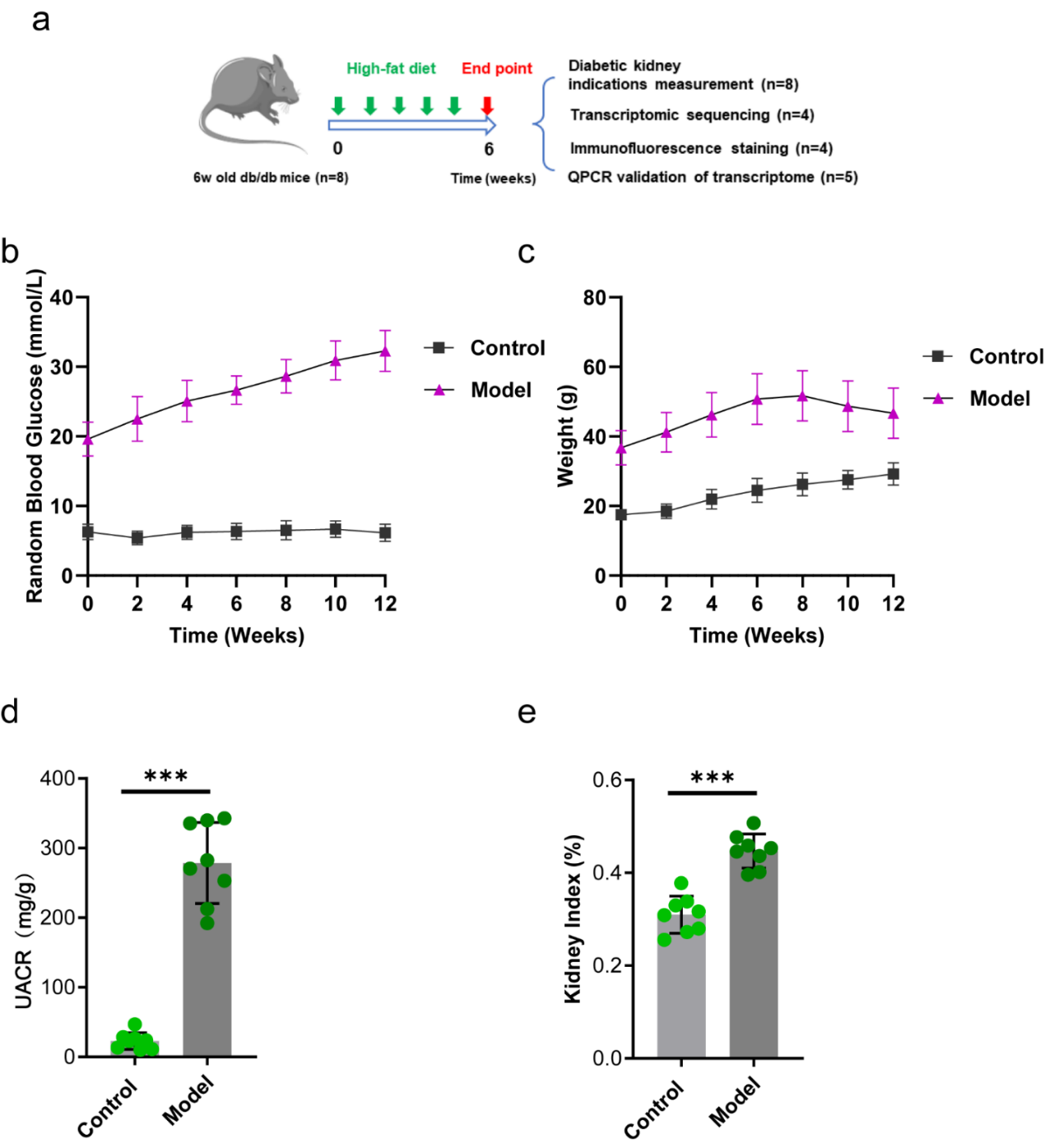


**Supplementary Figure 1. Establishment of diabetic nephropathy mouse model.** **(a)** Schematic diagram of establishing diabetic nephropathy mouse model. **(b)** blood glucose of diabetic mice. n=8. **(c)**Weight gain curve of diabetic mice. n=8. **(d)** UACR of diabetic mice. n=8. **(e)** Kidney wight to body weight of diabetic mice. n=8. ***p<0.001. Error bars indicate SD.


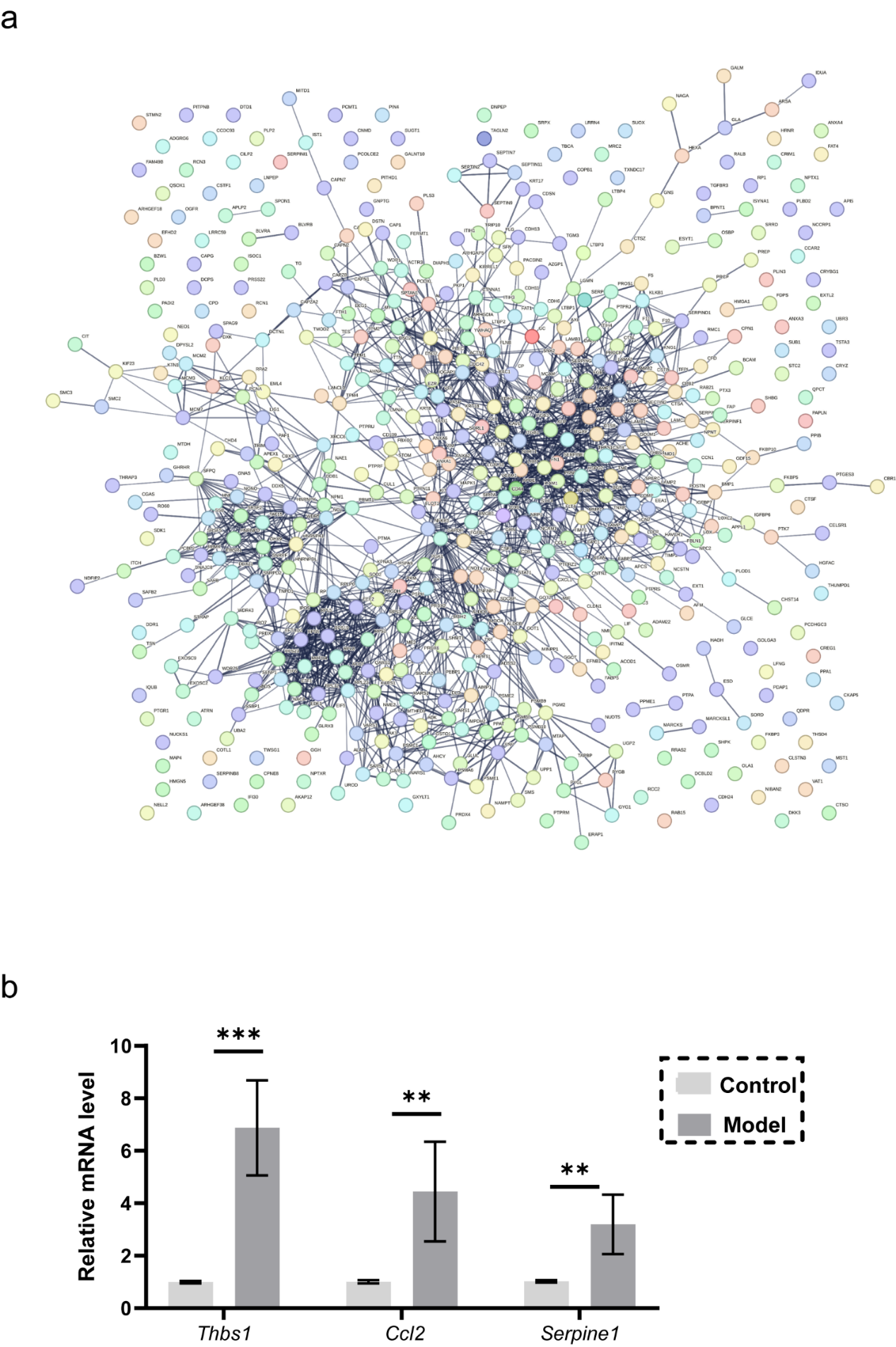


**Supplementary Figure 2. Analysis of core pathogenic proteins.** **(a)** PPI network of co-regulated proteins in transcriptome and proteome. **(b)** QPCR validation of core PPI proteins THBS1, CCL2, and SERPINE1. n=5. **p<0.01, ***p<0.001. Error bars indicate SD.


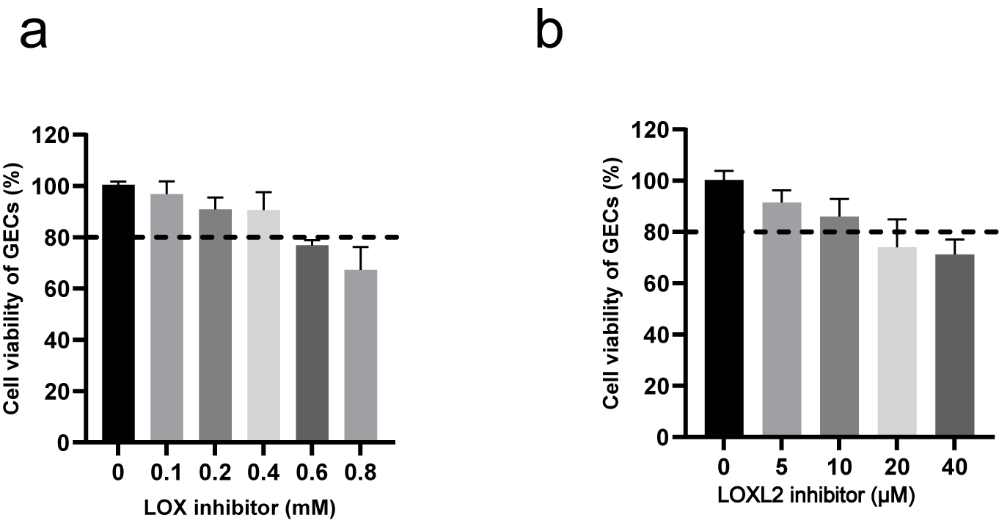


**Supplementary Figure 3. Cytotoxicity of LXO family inhibitors.** **(a)** BAPN cell viability assay. **(b)** PAT cell viability assay. n=4. Error bars indicate SD.


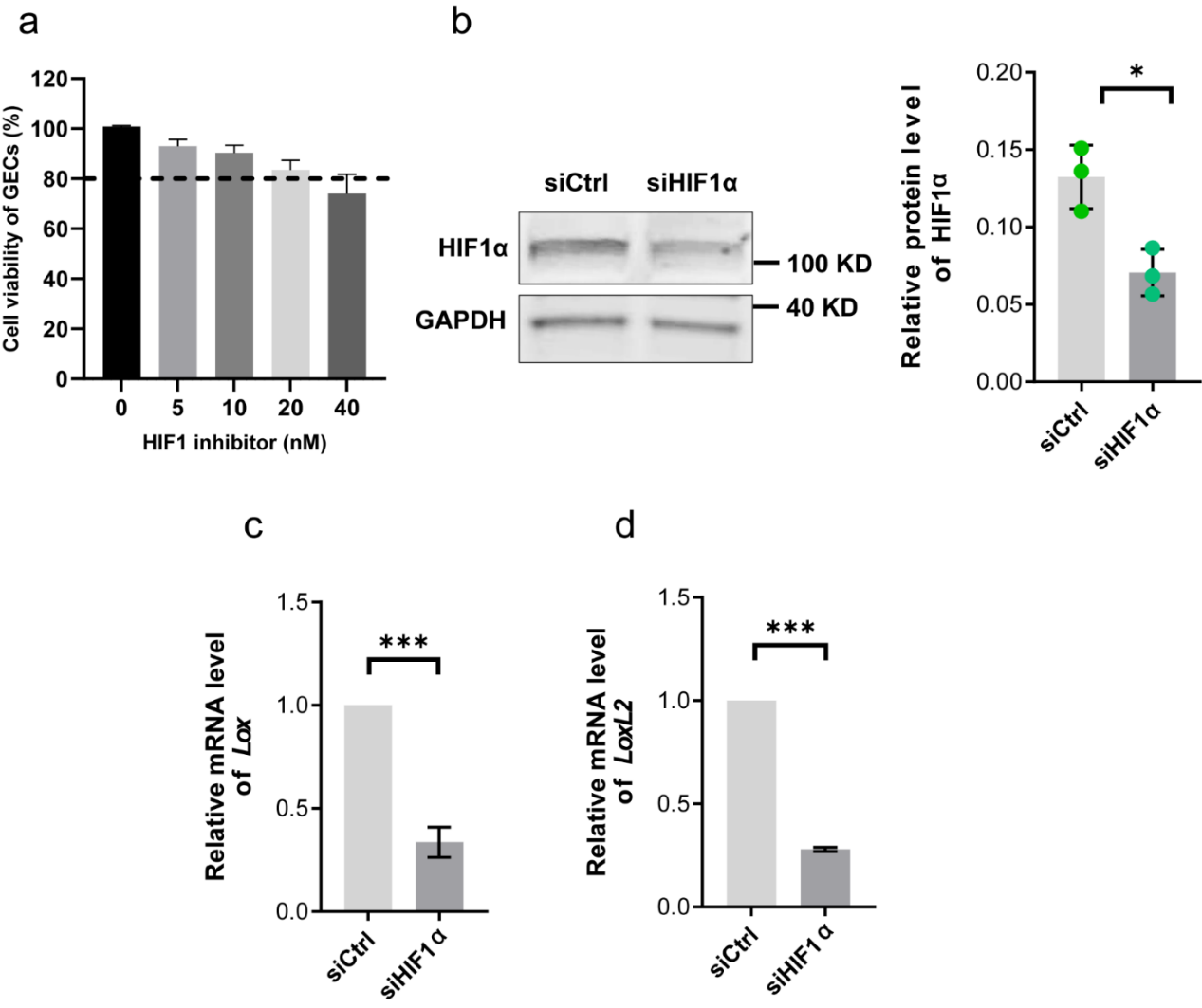


**Supplementary Figure 4. Regulatory effect of HIF1α on LOX family protein expression.** **(a)** Cytotoxicity of HIF1α inhibitor. **(b)** Protein level validation of HIF1α siRNA knockdown efficiency. n=3. Changes in **(c)** LOX and **(d)** LOXL2 mRNA levels after HIF1α siRNA transfection. n=3. *p<0.05, ***p<0.001. Error bars indicate SD.


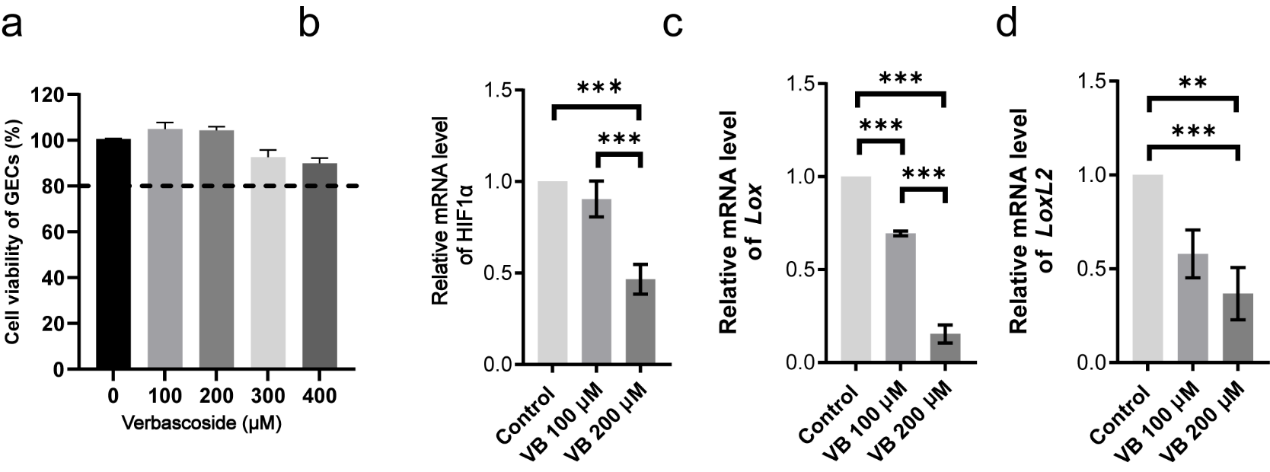


**Supplementary Figure 5. Effect of Verbascoside on the expression of HIF1α, LOX and LOXL2 mRNA levels.** **(a)** Verascoside cytotoxicity assay. n=4. **(b)** Changes in HIF1α mRNA levels. n=3. **(c)** Changes in LOX mRNA levels. n=3. **(d)** Changes in LOXL2 mRNA levels. n=3. **p<0.01, ***p<0.001. Error bars indicate SD.


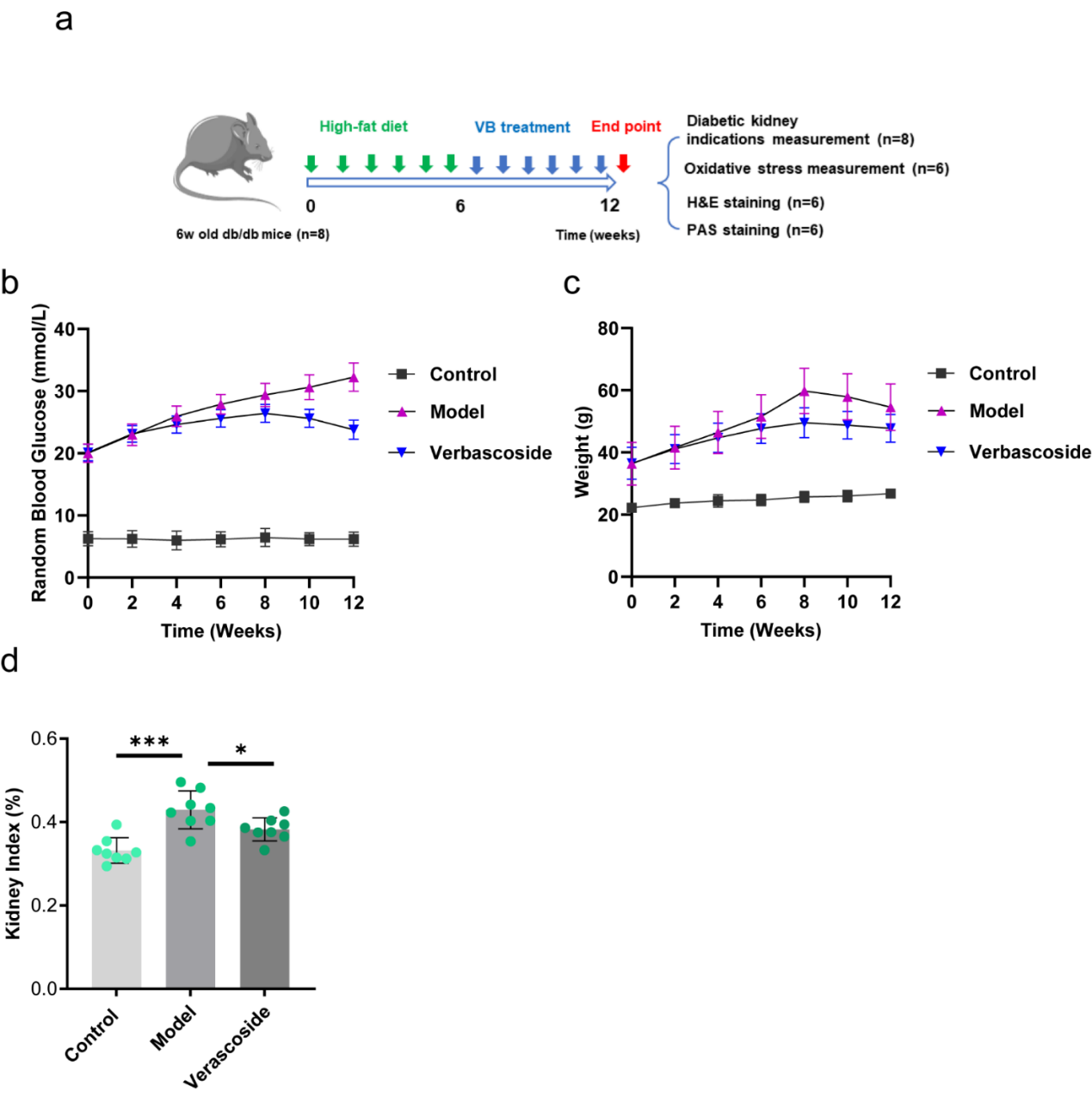


**Supplementary Figure 6.** **Verascoside improves renal functions in mice with diabetic nephropathy.** **(a)** Experimental flow chart of Verbascoside treatment of diabetic kidney mice. **(b)** Blood glucose levels in each group of mice treated with verbascoside. n=8. **(c)** Changes in body weight in each group of mice treated with verbascoside. n=8. **(d)** Changes in kidney weight to body weight in each group of mice treated with verbascoside. n=8. *p<0.05, ***p<0.001. Error bars indicate SD.
